# Artificial Intelligence Assists in the Early Identification of Cardiac Amyloidosis

**DOI:** 10.3390/jpm14060559

**Published:** 2024-05-24

**Authors:** Courtney R. Kenyon, Milagros Pereyra Pietri, Julie L. Rosenthal, Reza Arsanjani, Chadi Ayoub

**Affiliations:** Department of Cardiovascular Medicine, Mayo Clinic, Phoenix, AZ 85054, USA; kenyon.courtney@mayo.edu (C.R.K.); pereyra.milagros@mayo.edu (M.P.P.); rosenthal.julie@mayo.edu (J.L.R.); arsanjani.reza@mayo.edu (R.A.)

**Keywords:** artificial intelligence, electrocardiography, echocardiography, MRI, cardiac amyloidosis, case report

## Abstract

A 69-year-old female presented with symptomatic atrial fibrillation. Cardiac amyloidosis was suspected due to an artificial intelligence clinical tool applied to the presenting electrocardiogram predicting a high probability for amyloidosis, and the subsequent unexpected finding of left atrial appendage thrombus reinforced this clinical suspicion. This facilitated an early diagnosis by the biopsy of AL cardiac amyloidosis and the prompt initiation of targeted therapy. This case highlights the utilization of an AI clinical tool and its impact on clinical care, particularly for the early detection of a rare and difficult to diagnose condition where early therapy is critical.

## 1. Introduction

AL amyloidosis is a rare condition characterized by the extracellular deposition of light chain protein fibers, also known as amyloid. Amyloid deposits can involve multiple organs, including, but not limited to, kidney, heart, gastrointestinal tract, nerves, and skin. The diagnosis of AL cardiac amyloidosis can be challenging and requires clinical expertise, and there is often a large testing burden [1,2]. A delay in its diagnosis is often a barrier to accessing appropriate care, and in the case of AL cardiac amyloidosis, which can be rapidly progressive and fatal, early diagnosis is critical [3,4]. Artificial intelligence applied to ECG for the screening and early detection of cardiac amyloidosis has recently been validated [5,6].

## 2. Clinical Case

A 69-year-old female with a medical background of ulcerative colitis status post remote ileostomy presented to the Emergency Department with shortness of breath and fatigue for 3 weeks. She was found to be in new onset atrial fibrillation (AF) with rapid ventricular response. A physical examination demonstrated volume overload with 1+ bilateral ankle pitting edema, jugular venous distension, and bilateral crepitations at the lung bases.

**Figure 1:**
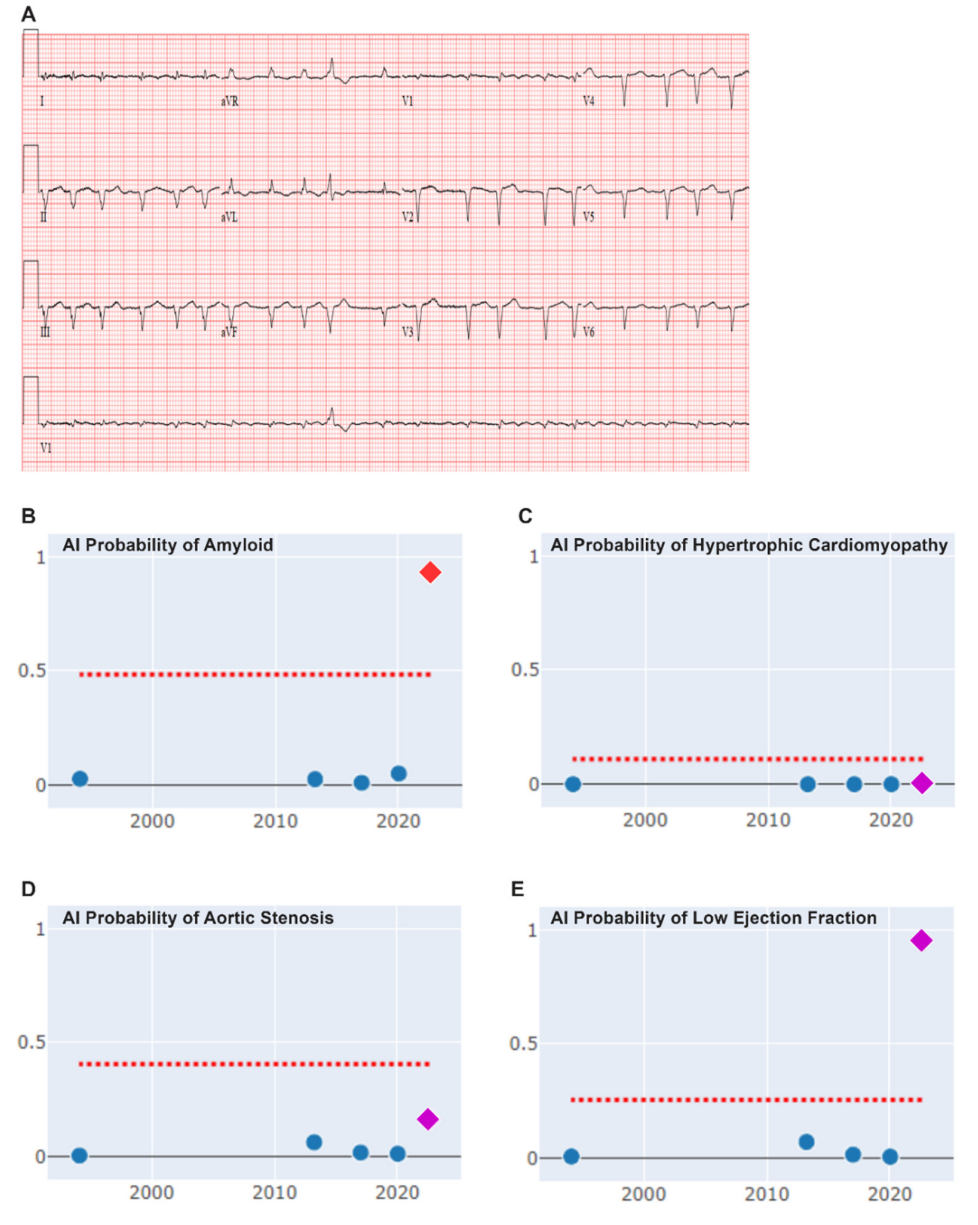
Artificial intelligence results applied to Electrocardiogram (ECG). Panel (**A**) demonstrates twelve lead ECG with atrial fibrillation with rapid ventricular response (117 beats per minute), low voltage, and pseudoinfarct pattern. Panel (**B**) shows artificial intelligence (AI) increased probability for cardiac amyloidosis at 93% (red diamond) together with 0.2% probability for hypertrophic cardiomyopathy (panel (**C**), purple diamond), 17% probability for aortic stenosis (panel (**D**), purple diamond), and 89% probability of low ejection fraction (panel (**E**), purple diamond). In panels (**B**–**E**), the blue dots represent previous ECG AI prediction for the respective conditions, and the red dotted line represents the cut off increased probability for that condition as predicted by AI.

Electrocardiogram (ECG) demonstrated AF with rapid ventricular response (117 (bpm), low QRS voltage, and pseudo-infarct pattern. Artificial intelligence (AI) ECG analysis flagged an increased likelihood of amyloidosis (at 93.1% probability) (Figure 1). Laboratory cardiac biomarkers included mildly elevated troponin T (24 ng/L, reference value < 10 ng/L) without change on serial measurement and elevated NT-proBNP (2609 pg/mL, reference value < 540 pg/mL).

**Figure 2:**
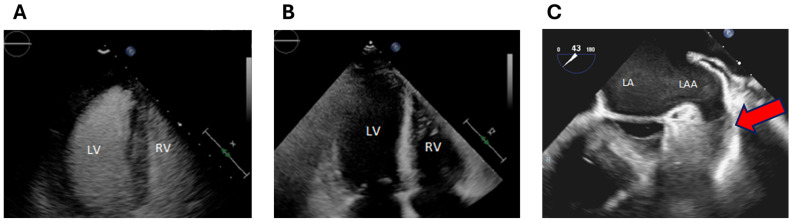
Imaging with Echocardiography. Panels (**A**,**B**) are transthoracic echocardiogram (TTE) apical four chamber views with and without ultrasound enhancing agent, respectively, showing borderline increased left ventricular wall thickness. Panel (**C**) is a transesophageal echocardiogram image demonstrating left atrial appendage with thrombus (red arrow) and spontaneous echo contrast in the left atrium. Abbreviations: LV—left ventricle, RV—right ventricle, LA—left atrium, LAA—left atrial appendage.

Transthoracic echocardiogram (TTE) demonstrated normal left ventricular ejection fraction (EF) of 55% and borderline increased concentric LV wall thickness (septal and posterior wall thickness 12 mm by echo enhancing agent measurement) (Figure 2A,B). LV filling pressures were elevated with E/e’of 28, and there was bilateral atrial enlargement. There was no pericardial effusion. Strain measurement could not be performed due to suboptimal endocardial definition on unenhanced 2D echocardiography.

In the setting of a CHADSVASC2 score of 2 (for age, female sex), direct oral anticoagulant with apixaban was commenced. As AF appeared symptomatic for >48 h, transesophageal echocardiogram (TEE) was performed prior to the planned direct current cardioversion, which identified a large laminated left atrial appendage (LAA) thrombus 1.7 cm × 1.2 cm (Figure 2C). Cardioversion was cancelled.

**Figure 3:**
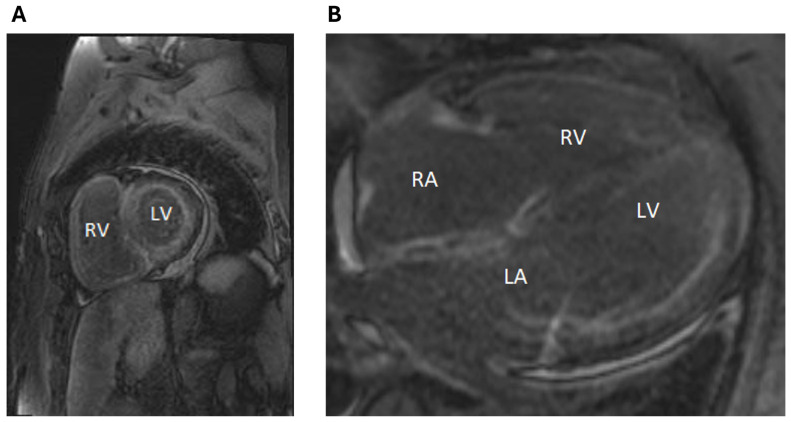
Cardiac Magnetic Resonance Imaging (MRI) findings. Panels (**A**,**B**) are delayed-phase images on cardiac MRI in the short-axis, and 4-chamber views, respectively, demonstrating diffuse late gadolinium uptake throughout the left and right ventricular myocardium as well as in the atrial free walls, consistent with infiltrative process such as cardiac amyloid. Abbreviations: LA—left atrium, LV—left ventricle, RA—right atrium, RV—right ventricle.

Cardiac magnetic resonance (CMR) imaging was performed to evaluate for suspected cardiac amyloidosis and demonstrated significant and diffuse late gadolinium enhancement throughout the left ventricle, right ventricle, and the walls of both atria, consistent with infiltrative process (Figure 3). Serum-free light chains (FLCs) were elevated (Kappa 13.7 mg/dL, lambda 1.61 mg/dL), with a kappa/lambda ratio of 8.51. Serum and urine protein electrophoresis with immunofixation showed no monoclonal protein or M spike.

Hematology was promptly consulted. The initial fat pad biopsy was negative for amyloid; however, the subsequent repeat fat pad biopsy was focally positive for amyloid deposition on Congo red stain. The bone marrow biopsy confirmed plasma cell neoplasm (kappa monotypic, approximately 10% plasma cells), and the Congo red stain was focally positive for amyloid in the small vessel walls. With a diagnosis of AL cardiac amyloid established, prompt therapy was initiated with Daratumumab and CyBorD (cyclophosphamide, bortezomib, and dexamethasone).

## 3. Discussion

This presentation highlights the translational application and clinical utility of artificial intelligence for the prediction of cardiovascular disease by ECG, particularly in rare conditions that are challenging to diagnose early such as cardiac amyloidosis. The patient’s presentation with symptomatic AF and congestive heart failure is common [7], and in itself would not have prompted further investigation for cardiac amyloid. The presence of a left atrial appendage thrombus, although clinically unexpected with a presumptive CHADSVASC2 score of 2, otherwise would have been anticoagulated and a rate control strategy pursued. Notably, once a diagnosis of cardiac amyloidosis is made, this population is considered to be at high risk of atrial thrombus formation, and anticoagulation would be necessary with AF, and the CHADSVASC 2 score would not be applicable [8,9,10,11,12].

This AI algorithm which can be applied in the electronic medical record (EMR) in our institution had flagged the presenting ECG as a high likelihood for cardiac amyloidosis. It served as a prompt even prior to the TEE identification of LAA thrombus. The presence of borderline increased thickness on TTE, low-voltage ECG with pseudoinfarct pattern, and heart failure are all supportive clinical features that are consistent with the diagnosis of cardiac amyloid. However, individually, they are not specific, and their common occurrence may not necessarily prompt clinicians to further evaluate for amyloidosis, particularly those without substantial experience with this uncommon condition.

The AI flag in conjunction with the identification of LAA thrombus and subtle clinical suspicion for cardiac amyloidosis triggered further evaluation. CMR with extensive diffuse LGE in the myocardium confirmed the presence of an infiltrative process, and the degree of left atrial wall infiltration accounts for the LAA thrombus with left atrial contractile failure. Amyloidosis was confirmed by biopsy, permitting early targeted treatment, which is critical for prognosis [13].

AL amyloidosis is a rare condition characterized by the extracellular deposition of light chain protein and may affect multiple organs. Although its incidence has been increasing with time given increased awareness for its diagnosis, its prevalence has been estimated at 40.5 cases per million in the United States [14]. Therapy for AL amyloidosis with cardiac involvement aims to treat congestive heart failure, manage atrial fibrillation, and prevent the progression of amyloid [11]. AL amyloidosis-targeted therapy prevents the overproduction of light chains. The current standard of care includes daratumumab, a human CD38+ targeting therapy, and CyBorD; autologous stem cell transplant can be considered. Early diagnosis is critical, as AL cardiac amyloidosis carries 20% risk for mortality at 6 months if untreated. Prognosis is influenced by the success of hematologic response and degree of end organ involvement [11].

The diagnosis of AL cardiac amyloidosis can be challenging, and delay in diagnosis is often a barrier to accessing appropriate care [2]. Additionally, AL CA can be rapidly progressive with fatal outcomes if not detected in a timely fashion and targeted treatment such as chemotherapy not initiated promptly [3]. In a study of patients with AL amyloidosis, 37% did not have diagnosis established until after one year following the initial onset of symptoms, and the majority required visits to multiple different physicians in order to reach correct diagnosis [4]. TTE in this case had suboptimal endocardial definition and did not permit strain evaluation, in which an apical sparing pattern would be pathognomonic for CA and, if present, potentially prompts further evaluation. In addition to its rarity, patients with CA frequently present with nonspecific signs and symptoms, and given the variety of the diagnostic tests involved with variable sensitivity and specificity, the overall diagnosis of CA is often challenging [1,2].

In this context, the AI algorithm described in this case is useful to prompt the early identification of patients at an increased likelihood of having CA. It demonstrates the utility of AI in the detection of uncommon or rare diseases. The AI model was developed and trained using a large cohort consisting of 2997 patients, 60% of which were confirmed cases of ATTR or AL CA [5]. A deep neural network was trained on digital 12-lead ECGs within 180 days of diagnosis. The model architecture consisted of inputs containing raw ECG waveform data and a binary output predicting the presence or absence of CA. The AI model was tested on an internal validation set of 999 patients, and an optimal probability threshold of 0.485 was identified as the cutpoint for high versus low probability for CA [5]. The model had excellent performance for prediction on CA based on prediagnosis ECG, with area under the receiver operating curve (AUC) of 0.91, sensitivity of 0.84, and specificity of 0.85 [5]. The AI model had its post-development performance validated in a subsequent study of 440 patients with CA and 6600 controls, with an AUC of 0.84 [6]. The false positive rate of 15% and false-negative rate of 16%, respectively, while associated with excellent model performance, do mandate, nonetheless, the exercise of clinical judgment and standard of care clinical evaluation.

Further prospective studies are required to assess performance metrics for the ECG AI model in terms of a reduction in time to diagnosis and cost effectiveness. There are some potential limitations and challenges associated with the use of AI, including that the quality of the clinical ECG acquisition may affect the performance of AI models in the real-world setting. Although validated, this AI tool may be less generizable in some populations, such as Hispanic, given their smaller sample size in the training and validation cohorts [6]. The current technology requires the clinician to click on the AI function to derive prediction for CA on an ECG; in future, such technology may be automatically applied to ECGs and the generated ECG interpretation. However, the mass application of AI may introduce ethical or patient privacy considerations, particularly in flagging potential diseases that may affect coverage by insurance prior to confirmatory diagnostic testing. It must be noted that, presently, this is not a diagnostic tool, and such technology is useful for opportunistic screening for those with a higher risk for CA. Although it does not replace the need for clinical judgment and evaluation, it may reduce the burden of testing in low-risk individuals and identify high-risk individuals for earlier diagnosis who may benefit from the expedited institution of treatment [15].

In conclusion, an AI clinical predictive model for ECG in the EMR accurately flagged high probability of a rare condition that had subtle and early clinical manifestations and carries poor prognosis if untreated. The AI clinical tool facilitated dedicated evaluation early and the prompt initiation of disease-targeted therapy for AL cardiac amyloidosis.

## 4. Learning Objectives

- To recognize that AI applied to ECG has clinical utility for the early detection of rare and difficult-to-diagnose conditions such as AL cardiac amyloidosis.
- To recognize that the early diagnosis of cardiac amyloidosis is critical as it carries poor prognosis if untreated and requires the prompt initiation of targeted therapy.

## Data Availability

Data sharing is not applicable.

## Abbreviations

AL	light chain amyloidosis
AI	artificial intelligence
ECG	electrocardiogram
EF	ejection fraction
LV	left ventricle
TEE	transesophageal echocardiogram
MRI	magnetic resonance imaging
TTE	transthoracic echocardiogram
LGE	late gadolinium enhancement
EMR	electronic medical record
